# Recycling Alone or Protesting Together? Values as a Basis for Pro-environmental Social Change Actions

**DOI:** 10.3389/fpsyg.2018.01229

**Published:** 2018-07-26

**Authors:** Daniel Sloot, Maja Kutlaca, Vanja Medugorac, Petra Carman

**Affiliations:** ^1^Department of Psychology, Faculty of Behavioural and Social Sciences, University of Groningen, Groningen, Netherlands; ^2^Department of Social Psychology, University of Osnabrück, Osnabrück, Germany; ^3^University College Dublin, Dublin, Ireland; ^4^Ivo Pilar Institute of Social Sciences, Zagreb, Croatia; ^5^Braziers Park School of Integrative Social Research, Ipsden, United Kingdom

**Keywords:** social change, pro-environmental behavior, activism, individual values, collective action

## Abstract

Social change can be pursued by participating in a public protest, joining a community gardening initiative, or recycling at home. However, little research has investigated how individual differences in values relate to people’s engagement in different types of social change actions in the context of pro-environmental behavior. We hypothesized that values would be differentially related to different types of social change actions, based on different goals that each of these actions may have (e.g., changing one’s own behavior or influencing others). A survey among people engaged in pro-environmental activism during the 2015 United Nations Climate Change Conference supported our predictions. Specifically, we found that individual behavior and community-based actions were uniquely related to biospheric values (i.e., a key concern for nature and the environment). However, other social change actions (e.g., public protest) were uniquely related to altruistic values (i.e., a key concern for the welfare of all people), and pro-environmental lobbying was positively related to egoistic values (i.e., a key concern for power and achievement). Our findings suggest that different behaviors directed at pro-environmental social change may be based on different values. We discuss the theoretical and practical implications of these findings.

## Introduction

People pursuing social change can do so by engaging in different actions to support their cause: they can join a public protest, engage in lobbying to influence authorities, or change their own individual behavior, for example, by recycling or saving energy in their household. According to social psychological theories on social change, such actions are mainly driven by people’s attachment to and identification with significant groups (e.g., Simon and Klandermans, 2001; Van Zomeren et al., 2008). Importantly, recent views suggest that these group (or politicized) identities are moral identities (Kutlaca et al., 2017; Turner-Zwinkels et al., 2017), and that values play an important part in motivating social change (Skitka and Bauman, 2008; Van Zomeren, 2013; Mazzoni et al., 2015; Thomas et al., 2016). The integration of individual values into research on social change is important not only because values can explain *why* people act, but possibly also *which* actions people engage in to achieve social change.

Although values are considered to be universal guiding principles in people’s lives and thereby not bound by context (Rokeach, 1973; Schwartz, 1992), research suggests that people often fail to relate their important values to their actions (Maio et al., 2001, 2009). One explanation for this value-behavior gap is that some behaviors may be more typical expressions of a particular value than others (Maio, 2010). Building on this assumption, we propose that distinct types of actions aimed at social change can be seen as more or less typical expressions of different values. We examine this question in the context of pro-environmental social change. Previous work in this domain has focused on individual pro-environmental behavior in the household—such as recycling or domestic energy saving—and has established a link between these behaviors and biospheric values in particular (i.e., valuing nature and the natural environment; Steg et al., 2014a; Dietz, 2015). The question remains, however, why people might engage in other social change actions, such as public protest or civil disobedience, on behalf of the environment. By integrating research on individual values and social change, we aim to investigate the differential motivational basis for these actions among those who engage in pro-environmental social change.

### Different Pro-environmental Social Change Actions

People pursuing social change on behalf of a certain cause can choose between an array of different actions they can engage in to reach their aims (e.g., Wright, 2009). To illustrate, people can change their individual behavior by recycling or saving more energy in their household, which are often seen as typical examples of pro-environmental behavior. However, this household pro-environmental behavior is rarely studied in social change research, which mostly focuses on explaining individuals’ motivations to take part in collective actions such as public protests. Hence, though recent theorizing has acknowledged that ‘social change can arise from putting the thermostat at a lower level or participating in mass protest’ (Van Zomeren, 2014, p. 776), research has rarely examined and directly compared these different types of actions.

We propose that these pro-environmental social change actions do not only differ in whether they are pursued individually or with other people, but can be differentiated according to the goals they may fulfill. First, prior theoretical work on pro-environmental social change suggests that individuals’ commitment to the environment can be expressed by changing one’s own behavior, thereby ‘living the change one wants to see’ (Baggott, 1995; Lhotka et al., 2008). We label these actions as *self-directed* social change actions (Saunders, 2013) because they are directed at changing individual behavior without primarily aiming to change the behavior of other people. Self-directed change can be expressed by changing one’s individual (household) behavior or engaging in community-based initiatives such as community gardening or local exchange trading schemes. Second, individuals may also engage in actions that pursue broader societal change by influencing others (Simon and Klandermans, 2001). This can be achieved by engagement in what we call *other-directed* actions, primarily directed at influencing or changing behavior of other people. Other-directed social change actions can range from normative public protest and non-normative forms of civil disobedience to pro-environmental lobbying (Simon and Klandermans, 2001; Hornsey et al., 2006; Tausch et al., 2011). Both self-directed and other-directed actions may address the same ultimate cause (i.e., protecting the environment), and people committed to this cause might perform all of them to some extent. Although self-directed social change actions may provide more direct benefits for the individual (such as saving money by saving energy) than other-directed actions, we see the key difference in that self-directed change enables individuals to focus on personal change and act directly in line with their beliefs, whereas other-directed behaviors enable people to reach out to and influence others to change their behavior. We propose that values play an important role in motivating individuals’ choices to engage in different social change actions, depending on their respective goal (i.e., self-directed or other-directed change).

### Individual Values and Pro-environmental Social Change Actions

In his theory of basic individual values, Schwartz (1992) proposed that values form a motivational continuum and can be ordered on two main polar axes. The first of these axes distinguishes openness to change values (emphasizing stimulation and independence) from conservation values (emphasizing tradition and conformity). The second axis contrasts self-enhancement (corresponding to self-interest or egoism) with self-transcendence (corresponding to altruism). Self-transcendence values express concern for social equality and caring for the welfare of all people, including a concern for the environment; in contrast, self-enhancement values express concern for personal status, power, and achievement (Schwartz, 1992; Schwartz et al., 2012). Self-enhancement and self-transcendence values were suggested as particularly relevant motivations inhibiting or promoting people’s engagement in pro-environmental behavior (De Groot and Steg, 2008; Dietz, 2015).

Steg and colleagues proposed a value scale specific to pro-environmental concerns that distinguishes between biospheric, altruistic, egoistic, and hedonic values (De Groot and Steg, 2008; Steg et al., 2014b; see also Stern et al., 1998). In their model, biospheric values emphasize a concern for nature and the natural environment, whereas altruistic values reflect a concern for social justice and the welfare of others (resembling Schwartz’ dimension of universalism). Egoistic values reflect a concern for personal status, power, and influence over others (resembling the power and achievement dimensions in Schwartz’ scale), and hedonic values emphasize enjoyment and positive feelings. An array of studies has provided compelling evidence that biospheric values motivate engagement in pro-environmental behavior (Schultz et al., 2005; Steg et al., 2011; Van der Werff et al., 2013; Van der Werff and Steg, 2016). Other studies have found a similar positive link between general self-transcendence values (encompassing biospheric and altruistic values) and different pro-environmental behaviors (Stern et al., 1999), but the unique effect of altruistic values tends to disappear when biospheric values are considered simultaneously (Van der Werff and Steg, 2016). In contrast, egoistic and hedonic values often do not promote—and may even inhibit—pro-environmental behavior (Dietz, 2015).

Importantly, research on the relationship between values and pro-environmental behavior has commonly focused on individual behavior (i.e., a type of self-directed social change) typically performed in one’s household, such as recycling or saving energy (Schultz et al., 2005; Steg et al., 2011; Van der Werff et al., 2013; Van der Werff and Steg, 2016), and has rarely examined other types of self- and other-directed behaviors people may engage in. For example, self-directed change actions can also be undertaken as part of a group, through community-based action, which is a type of small-scale group action that includes the wider community (Hargreaves et al., 2013). Thus, we predict that both individual pro-environmental behavior and community-based actions will be motivated by biospheric values only, as they allow individuals to fulfill their own personal environmental goals and ‘live the change they want to see.’

In contrast, research on other-directed pro-environmental change actions is scarce. For example, Stern and colleagues found a weak relationship between altruistic values and public protest (one type of other-directed change actions), but did not explicitly compare different other-directed behaviors with self-directed ones (Stern et al., 1999). Since biospheric values emphasize a concern for nature and the environment, we expect them to relate to *any* type of action ultimately addressing this concern, including other-directed social change actions. At the same time, we propose that altruistic values should play a more important role in motivating other-directed actions. Namely, by engaging in other-directed social change actions, individuals can facilitate broader societal changes. Importantly, many environmental issues cannot be solved without addressing broader problems such as social inequality or poverty (Posner and Weisbach, 2010), and other-directed behaviors like public protest and civil disobedience on behalf of the environment are often connected to such social justice issues. Furthermore, public protests can serve the goal to build stronger bonds between the participating activists and the broader public or potential followers (Simon and Klandermans, 2001; Hornsey et al., 2006). Therefore, we expect these actions to be uniquely and more strongly linked to altruistic values, as they more directly reflect such a concern for social justice. In addition to these more common forms, we also explore the motivations for pro-environmental lobbying, a behavior that has hardly been investigated as a form of social change (Wright, 2009). Pro-environmental lobbying is somewhat different from actions such as public protest, because it involves influencing others in a system of hierarchies and power structures in order to reach compromises that benefit the environment. On the one hand, pro-environmental lobbying may be seen as connected to social justice issues, thereby expressing altruistic values in particular. On the other hand, these actions may be related to egoistic values as they are associated with power and influencing others.

In order to examine our question of how distinct types of environmental action may be differentially associated with biospheric, altruistic, egoistic, and hedonic values, we surveyed environmental activists who were engaged in events related to the 2015 United Nations Climate Change Conference (COP21). This group provided a unique opportunity to investigate the relationship between values and behaviors, because these individuals are more likely to engage in a broader range of behaviors, including both self-directed and other-directed forms of action. At the same time, they are likely to hold particularly strong biospheric values, which may make it more likely to detect any unique influences of other value orientations on different social change actions.

## Materials and Methods

### Participants and Procedure

Participants were recruited during demonstrations and events related to the COP21. These included demonstrations at the conference in Paris itself and the COP21 Climate March in Amsterdam. Participants were asked to participate in an online study on environmental activism, and those who agreed received a link to the study via email a few weeks later. In total, 710 email addresses were collected in both locations, out of which 162 people responded by filling in the online survey upon being contacted. Due to the international character of this event, the questionnaire was translated to English, Dutch, and French by native speakers. The survey was part of a larger project on environmental activism and included various questions related to the COP event; only items relevant for this research question are described below. The sample ranged in age from 20 to 67 years old (*M* = 30.19; *SD* = 9.08). Out of the 76 participants who provided their background data, 57% were women and 86% of participants finished their bachelor studies. The participants came from more than 10 different European countries. Of the participants, 66% reported being an active member of an environmental organization for on average 4 years, indicating that our sample mostly consisted of highly educated and experienced international activists. The study received ethical approval from the ethical committee of the Psychology Department at the University of Groningen, and all participants gave their informed consent to participate in the online survey.

### Measures

#### Values

Sixteen items measured the endorsement of biospheric, altruistic, egoistic, and hedonic values as guiding principles in people’s life (Steg et al., 2014b). Each value was measured using a nine-point Likert scale from -1 (against my principles), 0 (not important at all) to 7 (extremely important) and research has shown this to be a valid and reliable way of assessing values in environmental contexts (Bouman et al., 2018). Biospheric values were measured with four items (e.g., Protecting the environment: preserving nature). Altruistic values also consisted of four items (e.g., Social justice: correcting injustice, care for the weak). Egoistic values were measured using five items (e.g., Social power: control over others). Hedonic values were measured with three items (e.g., Pleasure: joy, gratification of desires). See **Table 1** for descriptive statistics and scale reliabilities.

**Table 1:** Descriptive statistics (M, SD) and bivariate correlations between measures.

	α	*M*	*SD*	1	2	3	4	5	6	7	8
(1) Biospheric values	0.80	5.82	1.03								
(2) Altruistic values	0.76	5.95	0.92	0.47^∗∗^							
(3) Egoistic values	0.69	2.00	1.21	0.11	0.06						
(4) Hedonic values	0.82	4.36	1.43	0.15	0.19^∗^	0.21^∗^					
(5) Individual behavior	–	6.17	1.12	0.35^∗∗^	0.29^∗∗^	0.06	0.08				
(6) Community-based action	–	4.47	1.95	0.31^∗∗^	0.33^∗∗^	0.04	0.04	0.31^∗∗^			
(7) Public protest	–	5.36	1.33	0.12	0.24^∗∗^	0.06	-0.07	0.17	0.22^∗^		
(8) Civil disobedience	–	3.31	1.85	0.05	0.20^∗^	-0.20^∗^	-0.03	0.04	0.21^∗^	0.44^∗∗^	
(9) Pro-environmental lobbying	–	2.98	1.92	-0.04	0.07	0.27^∗∗^	-0.11	-0.04	0.06	0.33^∗∗^	0.09

^∗^*p < 0.05 (two-tailed); ^∗∗^*p* < 0.01 (two-tailed)*.

#### Self-Reported Social Change Actions

We constructed five single items to measure how often people had self-reportedly engaged in two different self-directed and three different other-directed actions over the past 12 months. First, participants were asked to rate self-directed actions: ‘Engaging in individual pro-environmental behavior (e.g., waste reduction, energy efficiency, green consumerism)’; ‘Engaging in community-based projects (e.g., community gardening, Transition movements, ecovillages, permaculture groups, local exchange trading schemes).’ Second, participants were asked to rate other-directed strategies: engaging in public protest was measured as ‘Campaigning for social change (e.g., participating in demonstrations, marches, street performances)’; ‘Civil disobedience and direct actions (occupying streets or buildings, disturbing events); pro-environmental lobbying was measured as ‘Consultation, dialog and compromise with authorities (e.g., lobbying, negotiating with and persuading governments and industry to take action on climate change).’^1^ Single items were used to keep the questionnaire relatively short and because all items reflected clearly described actual behaviors, as opposed to psychological constructs (Teixeira et al., 2011). All items were measured on a seven-point Likert scale from 1 (never), 4 (occasionally) to 7 (very frequently).

## Results

### Descriptive Analyses

The final sample used for the analyses consisted of 118 participants. We compared the value orientations of participants who did not fill in the social change action items (*N* = 44) to those who did; participants with missing answers only differed in that they endorsed egoistic values to a slightly higher extent (see Appendix A1). Participants strongly endorsed biospheric and altruistic values, while they placed little importance on hedonic and egoistic values (see **Table 1** for descriptive statistics and bivariate correlations). Regarding the social change actions, participants reported regular engagement in individual behavior but also in protesting for an environmental cause.

### Regression Analyses

In order to examine the value basis of different social change actions, we regressed each action separately on biospheric, altruistic, egoistic, and hedonic values (**Tables 2**, **3**).^2^ On average, values explained about 10% of the variance in each social change action. First, we looked at the association between values and self-directed social change actions. Supporting our hypothesis, only biospheric values were uniquely associated with individual pro-environmental behavior (**Table 2**). Similarly, there was a (marginally) significant unique relationship between biospheric values and community-based pro-environmental action. Interestingly, altruistic values were uniquely related to community-based action next to biospheric values (**Table 2**). Second, we looked at the association between values and other-directed social change actions. Contrary to expectations, biospheric values were not significantly related to public protest, civil disobedience, or pro-environmental lobbying. However, altruistic values were uniquely related to public protest and civil disobedience, supporting our hypothesis (**Table 3**). Interestingly, we found egoistic values to be negatively related to civil disobedience as well, suggesting that people engage in civil disobedience the more they endorse altruistic values and the less strongly they endorse egoistic values (**Table 3**). Lastly, we examined the relationship between values and pro-environmental lobbying. Neither biospheric nor altruistic values were linked to this action. Instead, egoistic values were uniquely and *positively* associated with pro-environmental lobbying, whereas hedonic values had a small negative relationship with this action.

**Table 2:** Regression models for self-directed social change actions (individual behavior and community-based actions).

	Individual behavior			Community-based actions		
	β	*t*	*p*	β	*t*	*p*
Biospheric values	0.27	2.70	0.008	0.20	1.98	0.050
Altruistic values	0.16	1.61	0.109	0.24	2.41	0.018
Egoistic values	0.02	0.27	0.789	0.01	0.13	0.898
Hedonic values	0.01	0.10	0.921	-0.03	-0.37	0.713

*F*		4.71			4.55		
*df*		4,113			4,113		
*p*		0.002			0.002		
*R^2^*_adjusted_		0.11			0.11		

**Table 3:** Regression models for other-directed social change actions (public protest, civil disobedience, and consultation with authorities).

	Public protest			Civil disobedience			Pro-environmental lobbying		
	β	*t*	*p*	β	*t*	*p*	β	*t*	*p*
Biospheric values	0.01	0.08	0.940	-0.03	-0.31	0.759	-0.11	-1.06	0.291
Altruistic values	0.26	2.52	0.013	0.24	2.29	0.024	0.14	1.35	0.181
Egoistic values	0.07	0.78	0.440	-0.21	-2.26	0.026	0.31	3.43	0.001
Hedonic values	-0.13	-1.42	0.159	-0.03	-0.26	0.793	-0.19	-2.02	0.046

*F*		2.39			2.77			3.75		
*df*		4,113			4,113			4,113		
*p*		0.055			0.031			0.007		
*R^2^*_adjusted_		0.05			0.06			0.09		

Since this last finding contradicts the common notion that egoistic values are commonly unrelated or even negatively related to pro-environmental behavior (e.g., Dietz, 2015), we decided to unravel this relationship further by exploratively looking at the relation between specific subcomponents of egoistic values and pro-environmental lobbying. Egoistic values as measured by Steg et al. (2014b) encompass different sub-dimensions in the theory of basic individual values (Schwartz, 1992; Schwartz et al., 2012): three items correspond to Schwartz’ sub-dimension of power (social power and authority to power-dominance; wealth to power-resources), and two items corresponded to the sub-dimension of achievement (influential and ambitious). Bivariate correlations between each of the five items in this scale and pro-environmental lobbying showed that the two items corresponding to the achievement sub-dimension were significantly positively correlated with this behavior (influential: *r*[116] = 0.21, *p* < 0.05; ambitious: *r*[116] = 0.29, *p* < 0.01), whereas the three items corresponding to the power sub-dimension were not (all *p*s > 0.05). We reflect on this finding in the discussion.

## Discussion

By integrating values with research on social change, this study investigated how biospheric, altruistic, egoistic, and hedonic values are associated with engagement in different social change actions on behalf of a pro-environmental cause. In sum, our findings suggest that different value orientations are related to engagement in different actions to pursue pro-environmental social change (see **Table 4** for a summary). First, we found that self-directed social change actions (both individual pro-environmental behavior and community-based actions) are uniquely related to biospheric values. This replicates previous findings on individual pro-environmental behavior (De Groot and Steg, 2008; Steg et al., 2014a), and extends previous research by investigating the less examined community-based actions. Taken together, this supports our hypothesis that self-directed social change is indeed concerned with ‘living the change one wants to see.’ In other words, these actions indeed seem to be the most typical expression of biospheric values. Importantly, we found that community-based actions were also uniquely related to altruistic values. It is possible that people engaged in community-based actions may in fact feel connected to bigger issues of social justice, although they commonly pursue the social change within their own community in a relatively self-directed way. The often-used slogan ‘Think global, act local’ captures the idea that locally confined actions may transcend the immediate goals of the community and include broader altruistic concerns.

**Table 4:** Summary of the relationships between values and social change actions.

Goal of social change action	Self-directed change		Other-directed change		
	
Action	Individual behavior	Community-based actions	Public protest	Civil disobedience	Pro-environmental lobbying
Biospheric	+	+	0	0	0
Altruistic	0	+	+	+	0
Egoistic	0	0	0	-	+
Hedonic	0	0	0	0	-

*+ Indicates a significant positive relationship; 0 indicates a non-significant relationship; 0 indicates a significant negative relationship*.

Second, we did not find a unique relationship between biospheric values and any other-directed social change actions. Though there is very little research on the relationship between biospheric values and environmental activism, this finding is surprising because biospheric values theoretically should relate to any type of actions ultimately aimed at benefiting the environment (De Groot and Steg, 2008). At the same time, this speaks to our reasoning that other-directed social change actions address both environmental and social justice concerns, and altruistic values play a central role in motivating such actions. Early theories of environmental behavior and activism conceptualized biospheric and altruistic values as a unidimensional construct (Stern et al., 1999). More recent research has distinguished these biospheric and altruistic value dimensions (De Groot and Steg, 2008). Our findings indicate that both dimensions can play an important role in explaining environmental social change, and while biospheric values motivate self-directed change, altruistic values are more relevant for engagement in political activism directed at influencing others. Another potential explanation for the missing link between biospheric values and other-directed social change actions could be the specific sample of seasoned activists examined in this study. Research by Hornsey et al. (2006) found that more experienced activists may perceive public protests as a possibility to build an oppositional movement, i.e., to reach out to a broader public and form a cohesive and strong activist movement. Thus, it is possible that these relatively highly engaged people may participate in protests more because they want to build a stronger network of activists that would address an unjust system. Thus, they may not see their actions directly linked to biospheric values (which they indeed strongly endorse), but rather altruistic values that reflect the need to address systemic problems.

Civil disobedience was not only linked to altruistic values, but also had a unique *negative* relationship with egoistic values. From a theoretical perspective, this fits the concept of egoistic values as expressing an importance of personal status, power, and ambition. While public protest (in Western countries) commonly takes place in organized settings, civil disobedience is a more extreme form of action that breaks existing social conventions or laws (Becker et al., 2011). Hence, whereas someone’s participation in a public protest does not lead to any negative personal consequences, engagement in civil disobedience bears a greater likelihood of punishment and loss of status and power.

Finally, pro-environmental lobbying was neither related to altruistic nor biospheric values, but was positively related to egoistic values. This finding is interesting, as research has provided little evidence for egoistic values to *promote* pro-environmental social change actions, though some have argued for a greater relevance of these values (Schultz and Zelezny, 2003; De Dominicis et al., 2017). Notably, our decomposition of the egoistic value scale to the item level revealed that endorsing values such as ‘ambitious’ and ‘influential’ was correlated with pro-environmental lobbying whereas endorsing values related to power or material status was not. Hence, it is not appreciation for material status or power that relate to people’s engagement in this social change action but rather their striving to influence other powerholders to support their cause. Future research should further examine the role of egoistic values, and it might be helpful to decompose the broad egoistic value orientation into its sub-dimensions (e.g., influence, ambition, etc.), as their positive or negative influence on pro-environmental actions might depend on the strength of the endorsement of these different sub-dimensions.

The average endorsement of the different values among the activists in our sample slightly differed from that of other samples in the literature. Our participants rated altruistic and especially biospheric values as more important compared to mean ratings in other studies, whereas they endorsed egoistic and hedonic values to a similar extent (e.g., Steg et al., 2011; Van der Werff and Steg, 2016). Other research on environmental activism also found that activists differ from general population. For example, environmental activists reported higher engagement in pro-environmental behavior and stronger endorsement of pro-environmental attitudes than the general population (Pahl et al., 2005). This is in line with other research on activism in general: activists are more likely to feel morally obliged to act against injustice (Sabucedo et al., 2018), and are more likely to moralize the issue (Skitka and Morgan, 2014). This suggests that they are more driven by their values and ideological beliefs than non-activists, and it would be interesting for future research to compare the motivations of both groups.

### Implications for Antecedents of Social Change Actions

For decades, scholars have investigated the antecedents of social change, aiming to identify the crucial factors that lead people to engage in behaviors such as public protest (Simon and Klandermans, 2001; Van Zomeren et al., 2008). In the domain of pro-environmental actions, a multitude of motivational factors have been proposed to promote such behaviors, including biospheric values, environmental self-identity, activist identity, and personal norms (Steg et al., 2014a; Van der Werff and Steg, 2016; Fritsche et al., 2017). In our view, the distinction between self- and other-directed pro-environmental behaviors actions may help future research disentangle which values and identities play a role. Due to the focus on personal change, self-directed behaviors may be more likely driven by an environmental self-identity (Van der Werff et al., 2013), a type of personal identity. In contrast, other-directed pro-environmental behaviors may rather be motivated by identification with a politicized group, e.g., a social movement organization such as Greenpeace (Simon and Klandermans, 2001; Van Zomeren et al., 2008). Moreover, our findings imply that different types of values may lie at the core of these identities: biospheric values may be more closely tied to environmental self-identity (Van der Werff et al., 2013), whereas the activist environmental identity may be more defined in terms of altruistic values. We hope that our study provides a promising basis for further research that could examine the relation between values, personal and group identities, and different types of social change actions.

### Limitations and Future Directions

It is a considerable strength of this study that it examined people engaged in environmental activism in a field setting. This sample is unique in that people are likely to engage in a variety of different actions related to pro-environmental behavior, including actions such as civil disobedience, which are less frequent among the general population. This provides the opportunity to study a range of social change actions together. At the same time, this means our sample is unlikely to be representative of the general population. Future research could address this by studying and comparing the motivations of non-activists with those engaging in activism, to gain a better understanding of why only some people translate their values into actions, especially those that are costly and risky behaviors. Furthermore, our research is correlational and we cannot draw any firm conclusions on the causality of our findings. Theorizing on values has conceptualized them as relatively stable, trans-situational factors that provide a basis for other motivational factors and guide behavior (Schwartz, 1992; Dietz, 2015). Nevertheless, the other direction is also possible: that is, people’s value responses may be based on their self-perceptions of the social change actions they engage in Maio and Olson (1998). Future research looking at long-term engagement in different forms of environmental behavior could provide insight into these processes.

### Practical Implications

It should be noted that our findings from this study are correlational, and future research is necessary to establish whether the found relationships are corroborated by experimental designs. This being said, our findings point to some interesting practical implications. First, scholars have suggested that any messages and campaigns aimed at environmental or social change should consider people’s underlying values to be effective (Corner et al., 2014). Our research supports this notion by showing that people’s engagement in different types of social change actions is predicted by different values. To give an example, practitioners campaigning for pro-environmental change can build their message in correspondence with biospheric values when aiming to promote self-directed change (e.g., motivating people to recycle). However, in order to effectively promote other-directed behaviors (such as public protest participation), it might be more effective to consider other types of values, particularly altruistic values. Hence, instead of only appealing to the importance of environmental protection, practitioners could put an emphasis on social justice or caring for other people, in order to more effectively motivate people to act. Moreover, a word of caution should be said about the potential use of egoistic values in campaigning for social change: while we find a positive relationship between egoistic values and consulting with authorities as one type of social change actions, research has suggested that appealing to such values may not promote, but often rather inhibit people from engaging in (pro-environmental) social change, as these instrumental values stand in contrast to the self-transcending values associated with social change actions (e.g., Evans et al., 2013; Dietz, 2015).

## Conclusion

In this paper, we examine how individual value orientations are uniquely related to different actions in the context of pro-environmental behavior. Our findings indicate that different values may play an important role in predicting different actions, including biospheric, altruistic, and even egoistic values. This may provide a fruitful basis for further theorizing and integration of theoretical accounts to explain people’s engagement in pro-environmental social change actions.

## Author Contributions

DS, MK, VM, and PC jointly designed the study and collected the data. DS and MK analyzed the data. DS wrote the paper, in conjunction with MK, and with input from VM and PC.

## Conflict of Interest Statement

The authors declare that the research was conducted in the absence of any commercial or financial relationships that could be construed as a potential conflict of interest. The reviewer BV and handling Editor declared their shared affiliation.

## Footnotes

1 Our distinction of the five social change actions into self-directed and other-directed actions was based on theoretical considerations. However, bivariate correlations between these measures showed preliminary support for this categorization (see **Table 1**). Specifically, individual pro-environmental behavior was only significantly related to community-based actions. While public protest was also positively related to community-based actions, in line with our categorization it was positively correlated with both civil disobedience and pro-environmental lobbying.

2 Since participants could answer the questionnaire in different languages, and were approached in both Paris and Amsterdam, we checked whether language or location had an influence on the results. For language, this was not the case. None of the regression models changed when language was added as a covariate. Location was related to public protest and civil disobedience: people approached in Paris were more likely to engage in these actions than those in Amsterdam. However, this only had a small effect on the regression model predicting civil disobedience, in which only egoistic values were a significant (negative) predictor when location was added as a covariate.

## Appendix

**Table A1:** Comparison between people with missing answers on all social change action items (dropped out) and those with filled-in answers (participated).

	Dropped out		Participated				
	*M*	*SD*	*M*	*SD*	*t*	*df*	*p*
(1) Biospheric values	5.89	0.99	5.82	1.03	0.41	160	0.682
(2) Altruistic values	5.69	1.09	5.95	0.92	-1.52	160	0.130
(3) Egoistic values	2.45	1.26	2.00	1.21	2.08	160	0.039
(4) Hedonic values	4.24	1.57	4.36	1.43	-0.46	160	0.646
